# Surgical management of “Twiddler syndrome” in patients with deep brain stimulation: a technical note and review of the literature

**DOI:** 10.1007/s00701-022-05135-8

**Published:** 2022-02-25

**Authors:** Z. Krause Molle, P. Slotty, J. Vesper

**Affiliations:** Dept. of Functional Neurosurgery and Stereotaxy, Neurosurgical Clinic, Heinrich Heine University, Düsseldorf, Germany

**Keywords:** Twiddler syndrome, Implanted impulse generator, Deep brain stimulation

## Abstract

**Background:**

The Twiddler syndrome (TS) describes a situation in which the implanted impulse generator (IPG) rotates several times around its own axis in the subcutaneous pocket. This can lead to severe mechanical damage of the leads and extensions and to dislocations.

**Method:**

Hereby, we report on a technique for revision surgery in patients diagnosed with Twiddler syndrome after undergoing previous deep brain stimulation (DBS) surgery. For revision surgery, the TYRX ™ Absorbable Antibacterial Envelope was used.

**Conclusion:**

The TS can be treated well with the envelope TYRX ™ Absorbable Antibacterial Envelope TYRX ™.

**Supplementary Information:**

The online version contains supplementary material available at 10.1007/s00701-022-05135-8.

## Description of technique

The Twiddler syndrome predominantly occurs in patients with CIED (cardiac implantable electronic devices). It describes multiple rotations of the pulse generator within the subcutaneous pocket, usually in combination with rolling up of the electrodes. This often leads to a malfunction of the system, either due to a breakage of the electrodes or due to an electrode dislocation. Clinically, a hypermobile stimulator as well as thickened cable strands or even cable pulls are palpable under the skin. A simple X-ray of the system can confirm the winding of the cables and is therefore sufficient to confirm the diagnosis.

The incidence of TS among implanted pacemakers is given as 0.07–1.1% [2]. The prevalence of Twiddler syndrome is reported to be 0.5–2.27% in patients with Deep Brain Stimulation (DBS) systems [7, 9] and 0.54–1.07% in patients with implanted SCS systems accordingly [1, 10].

It can develop spontaneously. Risk factors are an oversized implantation pocket or insufficient fixation. The possibility of unintentional manipulation by the patient is also discussed. Increasing risk factors for the occurrence of TS syndrome are female gender, higher patient age and an increased body mass index (BMI) [5].

In the present report, we illustrate a surgical approach using TYRX ™ Absorbable Antibacterial Envelope for revision surgery.

### Case 1

**Fig. 1:**
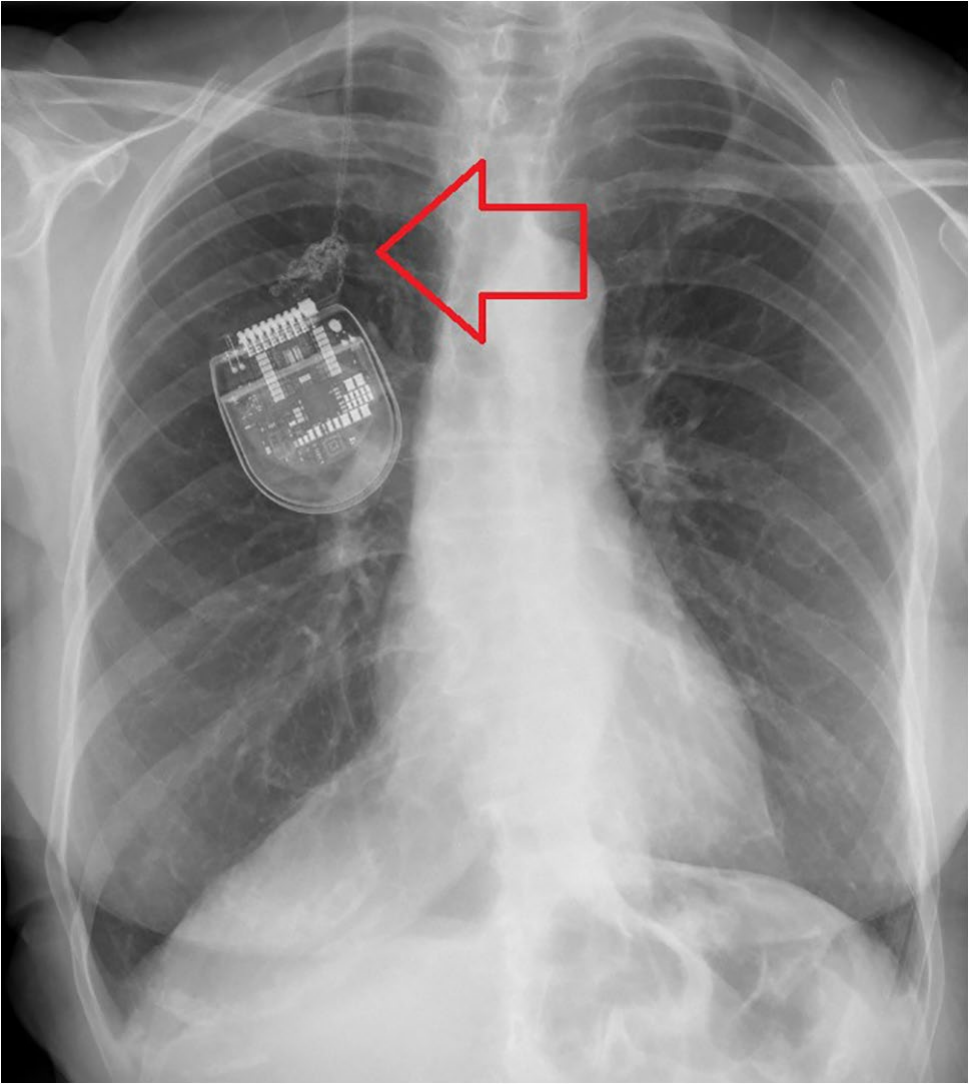
The thoracic X-ray (a.p.) shows the dislocation of the pectoral neurostimulator with twisted extensions (arrow)

A 75-year-old patient diagnosed with tremor was treated with a DBS system with bilateral VIM electrodes and a consecutive pectoral neurostimulator (Abbott, Infinity). One year later, the patient presented to our outpatient department with an increase in the previously well-suppressed tremor. High impedances were found on both electrodes throughout electronical check. Clinically, palpation of the stimulator showed hypermobilization and a significant thickening of the cables. Dislocation of the neurostimulator and an extension cable rolled-up several times was diagnosed by X-ray (Fig. 1).

### Intraoperative imaging

**Fig. 2:**
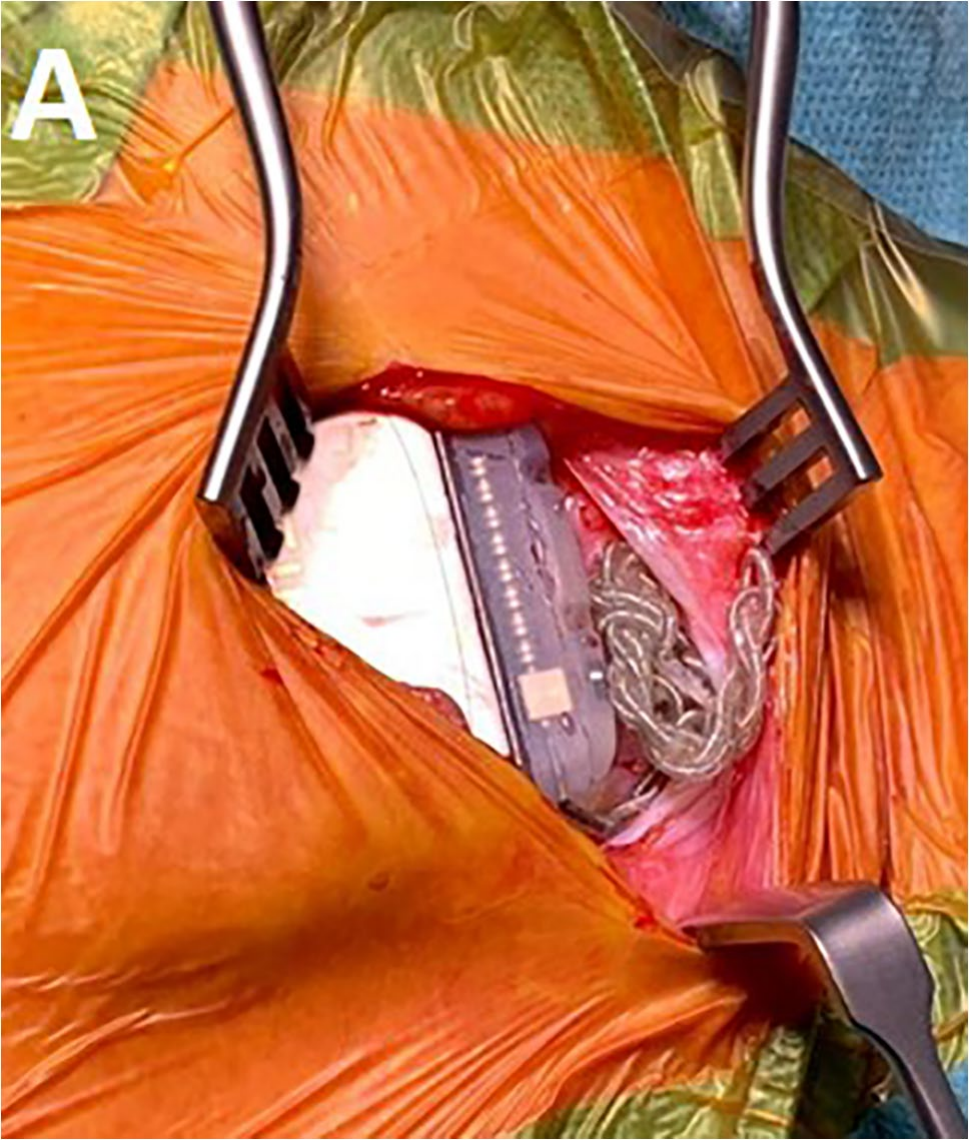
Intraoperative imaging of the twisted extensions in the IPG pocket

**Fig. 3:**
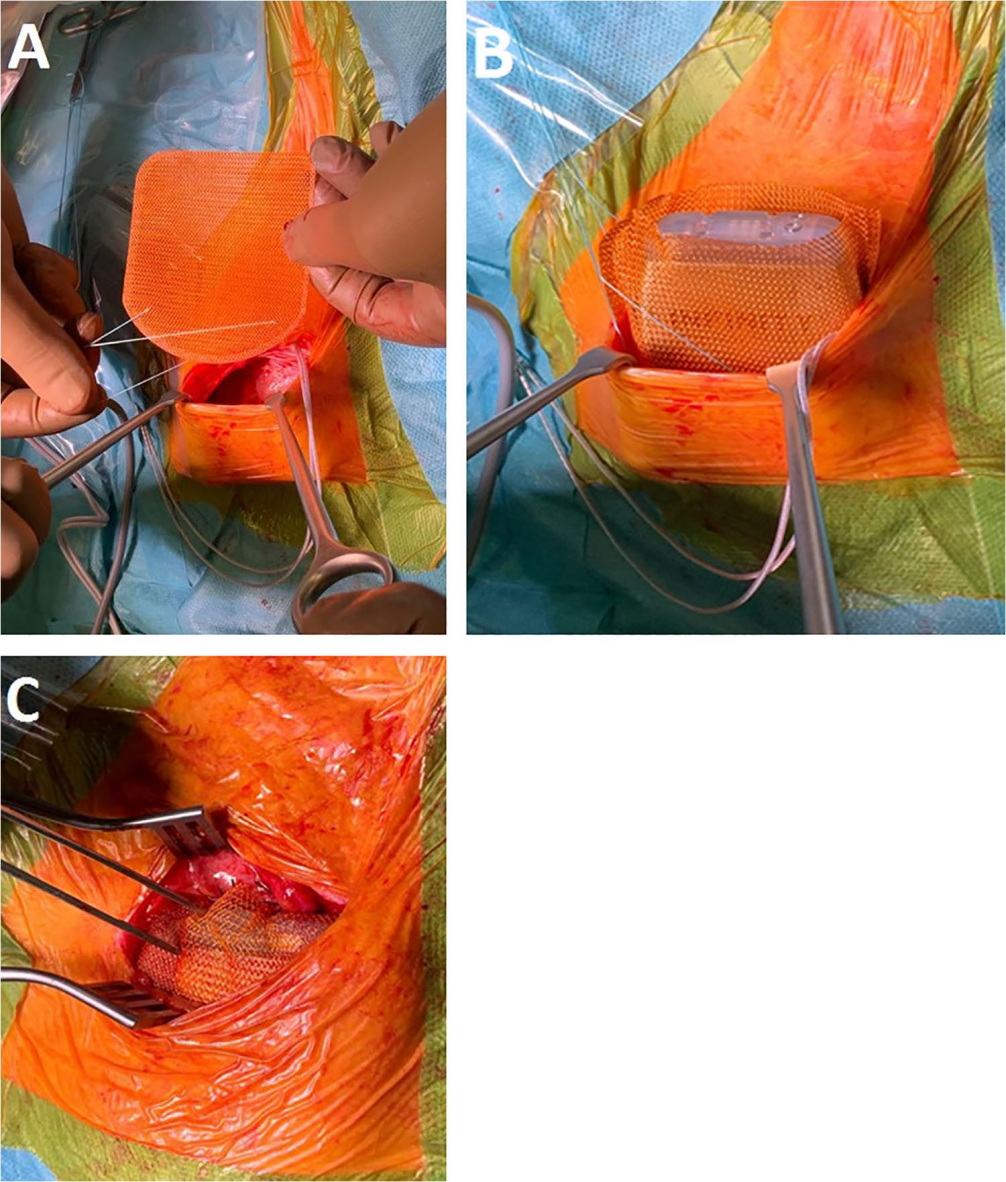
Intraoperative imaging with caudal fixation (**A**) and implantation of the TYRX ™ Absorbable Antibacterial Envelope with the device in the subcutaneous pocket (**B**). This is followed by the fixation of the neurostimulator with the Dacron bag on the cranial pole after the electrodes have been connected (**C**)

**Fig. 4:**
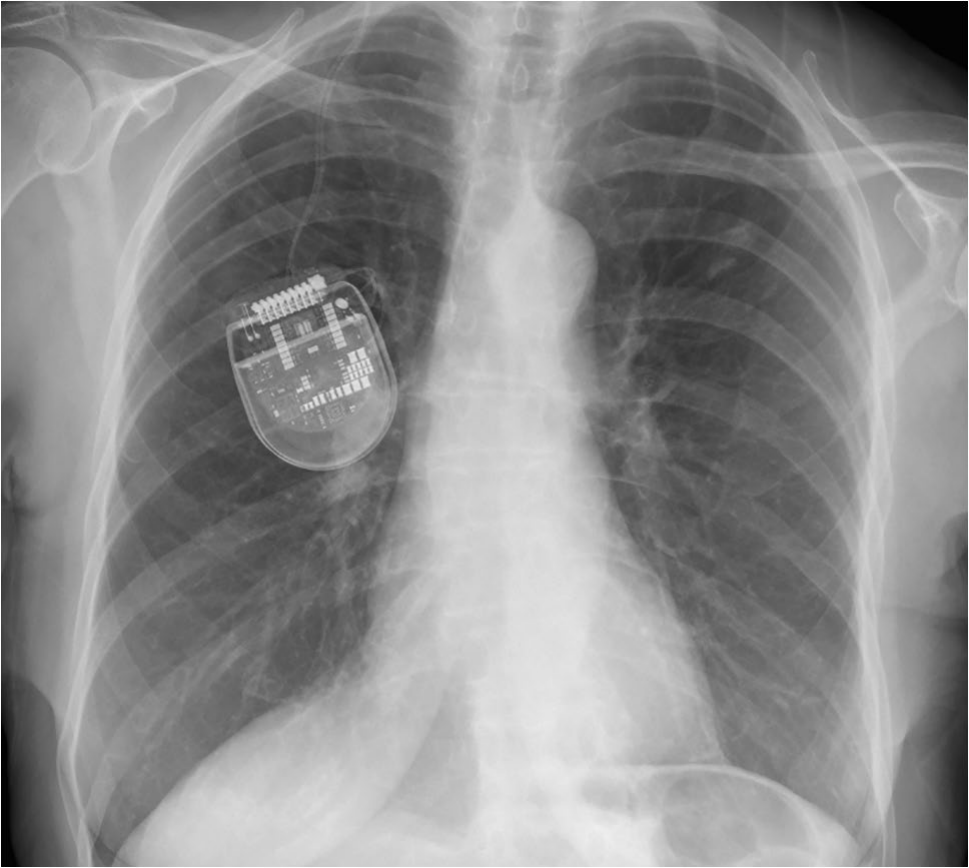
Postoperative chest x-ray with no signs of twisting of the electrodes

We performed revision surgery for fixation of the device. We therefore used a Dacron (TYRX ™ Absorbable Antibacterial Envelope, Medtronic TM) bag and additionally replaced the extension cables. The artificial pouch was fixed caudally by 2 non-absorbable sutures deep in the subcutaneous pocket on the fascia wall of the muscle. The neurostimulator was subsequently placed in the Dacron bag in the subcutaneous pocket. After connecting the extension cable to the neurostimulator, the Dacron bag was also fixed cranially to the fascia using the IPG holding devices with two non-absorbable threads (Figs. 2 and 3). An X-ray control at 6-month follow-up showed the expected result of a non-dislocated stimulator without any indication that the electrodes had been rewound (Fig. 4).

## Indication

Twiddler syndrome is a rare condition in patients with an implanted DBS system, yet no evidence-based statement for treatment is available. The published risk factors such as old age, female gender and increased body weight did not fully apply in our case. The abdominal or pectoral position of the neurostimulator also does not seem to have had any influence on the syndrome.

Our technique can be applied in patients with clinical and radiologically confirmed diagnosis of rotated pulse generators with consecutive atypical, twisted electrodes independent of underlying general diagnose.

## Limitations

Major limitation is, to date, the restricted access on data using Dacron pouches for fixation of IPGs. Due to limited publication on TS, it is not possible so far to identify a standard therapy concept based on a surgical recommendation for TS. Some of the current case reports recommended preventing mobilization of the neurostimulator during revision surgery, either with a smaller, adapted subcutaneous pocket or with an additional fixation suture [1, 3]. In 2017, Orsoro et al. were able to show that fixation of the device with a nonabsorbable antimicrobial pouch instead of just fixation sutures leads to a significant reduction in the recurrence rate (0% vs. 50%, *P*. < 0.05) in a retrospective data analysis of 23 patients with Twiddler syndrome in cardiac devices [8]. This is to our knowledge the largest cohort reported. One other case report was published on a patient with Twiddler’s syndrome with an implanted gastric electric stimulator, in whom the TYRX ™ Absorbable Antibacterial Envelope was already successfully used to stabilize the stimulator [4]. We also can only report on 2 cases in which the technique was used; hence, limited data is available for proof of concept.

## How to avoid complications

The use of TYRX ™ Absorbable Antibacterial Envelope to our knowledge is a safe option for patients with TS. Concerning required revision surgery and risk of postoperative infection, it offers additional safety due to release of antibiotics. It is a multifilament knitted mesh releasing minocycline and rifampicin for 7 days, which has been shown to lead to fewer postoperative infections without any additional relevant complications [6]. Furthermore, it is completely resorbed within 9 weeks after implantation.

Additionally, using a new pouch for replacement or revision surgery is recommend above all in patients with an increased BMI, especially if the neurostimulator has been implanted in the abdominal area. An abdominal fixation on the fascia would therefore be too deep to be able to guarantee telemetric control of the device.

## Patient information

The TYRX ™ Absorbable Antibacterial Envelope was developed for patients with CIED and an increased risk of postoperative infection. Data show that it can also be used safely and successfully in patients with twisted IPGs and consecutive non-functional twisted electrodes, for example in patients with TS.

## Supplementary Information

Below is the link to the electronic supplementary material.

Supplementary file1 (MP4 103679 KB)

## References

[1] Al-Mahfoudh R, Chan Y, Chong HP, Farah JO (2016). Twiddler’s syndrome in spinal cord stimulation. Acta Neurochir (Wien).

[2] Champagne C, *u. a.*, “Twiddler syndrome without lead dislodgment discovered by remote monitoring”, *Case Rep Cardiol*, Bd. 2021, Feb. 2021, 10.1155/2021/8816524. 10.1155/2021/8816524 PMC7884166 33628518

[3] Ghanchi H, Taka TM, Bernstein JE, Kashyap S, Ananda AK (2020). “The unsuccessful Twiddler: a case of Twiddler’s syndrome without deep brain stimulator lead breakage”. Cureus.

[4] Haslam M, Parkman HP, Petrov RV (2020). “Absorbable antibacterial envelope in the surgical management of Twiddler’s syndrome in a patient with gastric electric stimulator: a case report”. Dig Med Res.

[5] Kawata H, *u. a.*, “Obese female patients have higher rates of lead dislodgement after ICD or CRT-D implantation”, *Int J Cardiol*, Bd. 172, Nr. 3, S. e522–524, Apr. 2014, 10.1016/j.ijcard.2014.01.076. 10.1016/j.ijcard.2014.01.076 24502881

[6] Mittal S, *u. a.*, “The World-wide Randomized Antibiotic Envelope Infection Prevention (WRAP-IT) trial: long-term follow-up”, *Heart Rhythm*, Bd. 17, Nr. 7, S. 1115–1122, Juli 2020, 10.1016/j.hrthm.2020.02.011. 10.1016/j.hrthm.2020.02.011 32087357

[7] Morishita T, *u. a.*, “Postoperative lead migration in deep brain stimulation surgery: incidence, risk factors, and clinical impact”, *PLoS One*, Bd. 12, Nr. 9, Sep. 2017, 10.1371/journal.pone.0183711. 10.1371/journal.pone.0183711 PMC5597118 28902876

[8] Osoro M, Lorson W, Hirsh JB, Mahlow WJ (2018). Use of an antimicrobial pouch/envelope in the treatment of Twiddler’s syndrome. Pacing Clin Electrophysiol.

[9] Silva PA, Chamadoira C, Costa H, Linhares P, Rosas MJ, Vaz R (2014). Twiddler (or not) syndrome: questioning etiology for an uncommon form of hardware malfunction in deep brain stimulation. Surg Neurol Int.

[10] Son B, Choi J, Ha S (2018). Twiddler’s syndrome: a rare hardware complication in spinal cord stimulation. Asian J Neurosurg.

